# A simple additive-free approach for the synthesis of uniform manganese monoxide nanorods with large specific surface area

**DOI:** 10.1186/1556-276X-8-166

**Published:** 2013-04-11

**Authors:** Mingtao Zheng, Haoran Zhang, Xuebin Gong, Ruchun Xu, Yong Xiao, Hanwu Dong, Xiaotang Liu, Yingliang Liu

**Affiliations:** 1 Department of Applied Chemistry, College of Science, South China Agricultural University, Guangzhou, 510642, China

**Keywords:** Manganese monoxide, Nanorods, Additive-free synthesis, Formation mechanism

## Abstract

A simple additive-free approach is developed to synthesize uniform manganese monoxide (MnO) one-dimensional nanorods, in which only manganese acetate and ethanol were used as reactants. The as-synthesized MnO nanorods were characterized in detail by X-ray diffraction, scanning and transmission electron microscopy (TEM) including high-resolution TEM and selected-area electron diffraction, Fourier transform infrared spectrum, and nitrogen adsorption isotherm measurements. The results indicate that the as-synthesized MnO nanorods present a mesoporous characteristic with large specific surface area (153 m^2^ g^−1^), indicating promising applications in catalysis, energy storage, and biomedical image. On the basis of experimental results, the formation mechanism of MnO one-dimensional nanorods in the absence of polymer additives was also discussed.

## Background

During the past decade, manganese oxides have attracted considerable research interest due to their distinctive physical and chemical properties and potential applications in catalysis, ion exchange, molecular adsorption, biosensor, and energy storage [1-12]. Particularly, nanometer-sized manganese oxides are of great significance in that their large specific surface areas and small sizes may bring some novel electrical, magnetic, and catalytic properties different from that of bulky materials. A wide variety of manganese oxides (e.g., MnO_2_, Mn_2_O_3_, and Mn_3_O_4_) have been synthesized through various methods [13-24]. Among them, manganese monoxide (MnO) is a model system for theoretical study of the electronic and magnetic properties of rock salt oxides [25], and its nanoclusters interestingly exhibit ferromagnetic characteristics [26]. On the other hand, MnO is very interesting for its lower charge potential (1.0 V vs. Li/Li^+^) compared to other transition metal oxides [27]. It has been reported that a relatively high voltage and energy density can be obtained when it was coupled with a certain cathode material to construct a full lithium ion cell [28].

In terms of the synthesis methods of MnO, several approaches have been developed to prepare nanostructured MnO with different morphologies [28-42], such as hydrothermal reactions and subsequent annealing [28], thermal decomposition of Mn-containing organometallic compounds [29-32], thermal decomposition of MnCO_3_ precursor [33,34], vapor-phase deposition [37], etc. More recently, Lin et al. reported a simple one-pot synthesis of monodispersed MnO nanoparticles (NPs) using bulk MnO as the starting material and oleic acid as solvent [38]. Sun et al. reported a microwave-polyol process to synthesize disk-like Mn complex precursor that was topotactically converted into porous C-modified MnO disks by post-heating treatment [41]. However, these methods are often associated with the use of high-toxicity, environmentally harmful, and high-cost organic additives. Moreover, the by-products may have a detrimental effect on the size, shape, and phase purity of the MnO NPs obtained. It still remains a major challenge to prepare high-quality monophase MnO NPs due to the uncontrollable phase transformation of multivalent manganese oxides (MnO_2_, Mn_2_O_3_, and Mn_3_O_4_).

In the present work, we report a simple, cost-effective, and additive-free method for the synthesis of uniform MnO nanorods with large specific surface area, in which cheap manganese acetate and ethanol were used as starting materials. The microstructures of the as-synthesized products were investigated using scanning electron microscopy (SEM) and transmission electron microscope (TEM). The as-synthesized MnO nanorods present a mesoporous characteristic and large specific surface area. More importantly, we have avoided the use of expensive polymer or surfactant additives during the synthesis process. The possible formation mechanism for MnO nanorods in the absence of polymer additives was also discussed.

## Methods

### Preparation of MnO nanorods

In a typical synthesis, 1.0 g of manganese acetate was put into 30 mL of anhydrous ethanol distilled freshly to form a homogeneous solution under stirring. The solution was transferred to a 40-mL Teflon-lined stainless steel autoclave. These manipulations were operated in a glove box under N_2_ atmosphere. The autoclave was heated at 200°C for 24 h in an electric oven. After cooling to room temperature, the final products were washed with deionized water and ethanol several times and subsequently dried at 80°C for 6 h in vacuum.

### Instruments and characterization

The phase purity of the obtained samples was examined by X-ray diffraction (XRD) using an MSAL-XD2 X-ray diffractometer with CuKα radiation (*λ* = 0.15406 nm) operating at 40 kV and 20 mA. Morphologies of the samples were characterized by field emission scanning electron microscopy (JSM6700F). The morphology and structure of the MnO nanorods were further investigated by TEM and high-resolution transmission electron microscopy (HRTEM; JEM-2010, 200 kV) with energy-dispersive X-ray spectroscopy (EDS; INCA X200). X-ray photoelectron spectroscopy (XPS) was carried out by means of a Shimadzu AXIS UTLTRADLD spectrometer (Shimadzu, Kyoto, Japan). Nitrogen adsorption-desorption measurements were performed using a Micromeritics Tristar 3000 gas adsorption analyzer (Micromeritics Instrument Co., Norcross, GA, USA). Fourier transform infrared (FTIR) spectrum was measured by an Equinox 55 (Bruker, Ettlingen, Germany) spectrometer ranging from 400 to 4,000 cm^−1^.

## Results and discussion

**Figure 1:**
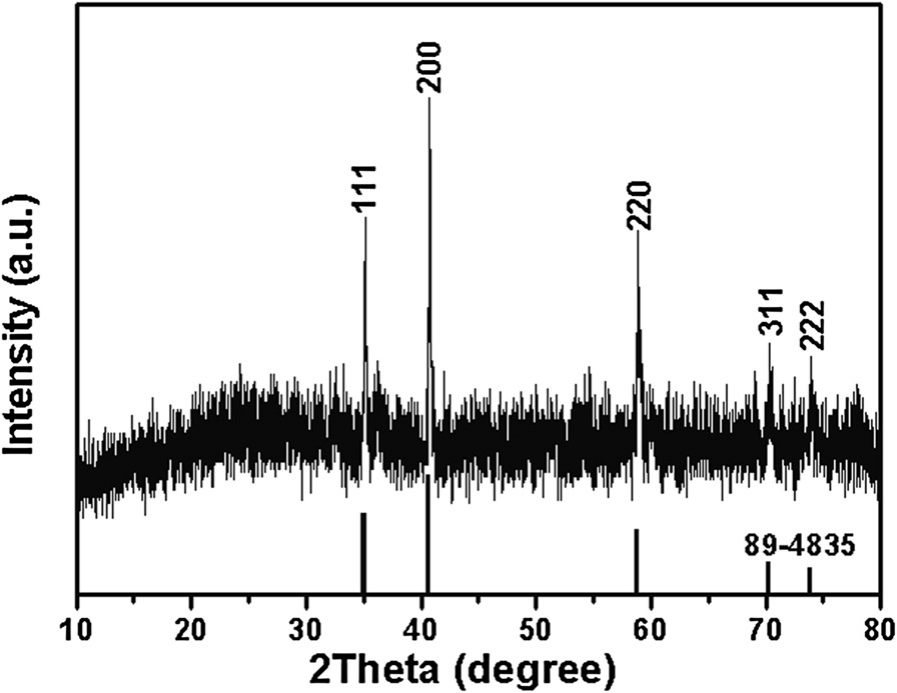
XRD pattern of as-prepared MnO nanorods synthesized at 200°C for 24 h.

Figure 1 shows the XRD patterns of the product synthesized at 200°C for 24 h. The diffraction peaks were observed at 2*θ* = 34.9°, 40.6°, 58.8°, 70.3°, and 73.8°, which could be assigned to (111), (200), (220), (311), and (222) reflections, respectively. These reflections could be readily indexed to cubic MnO with a lattice constant of 4.443 Å, in good accordance with the literature values (JCPDS 89–4835). No other phases of manganese oxide could be seen, indicating the monophase of cubic MnO.

**Figure 2:**
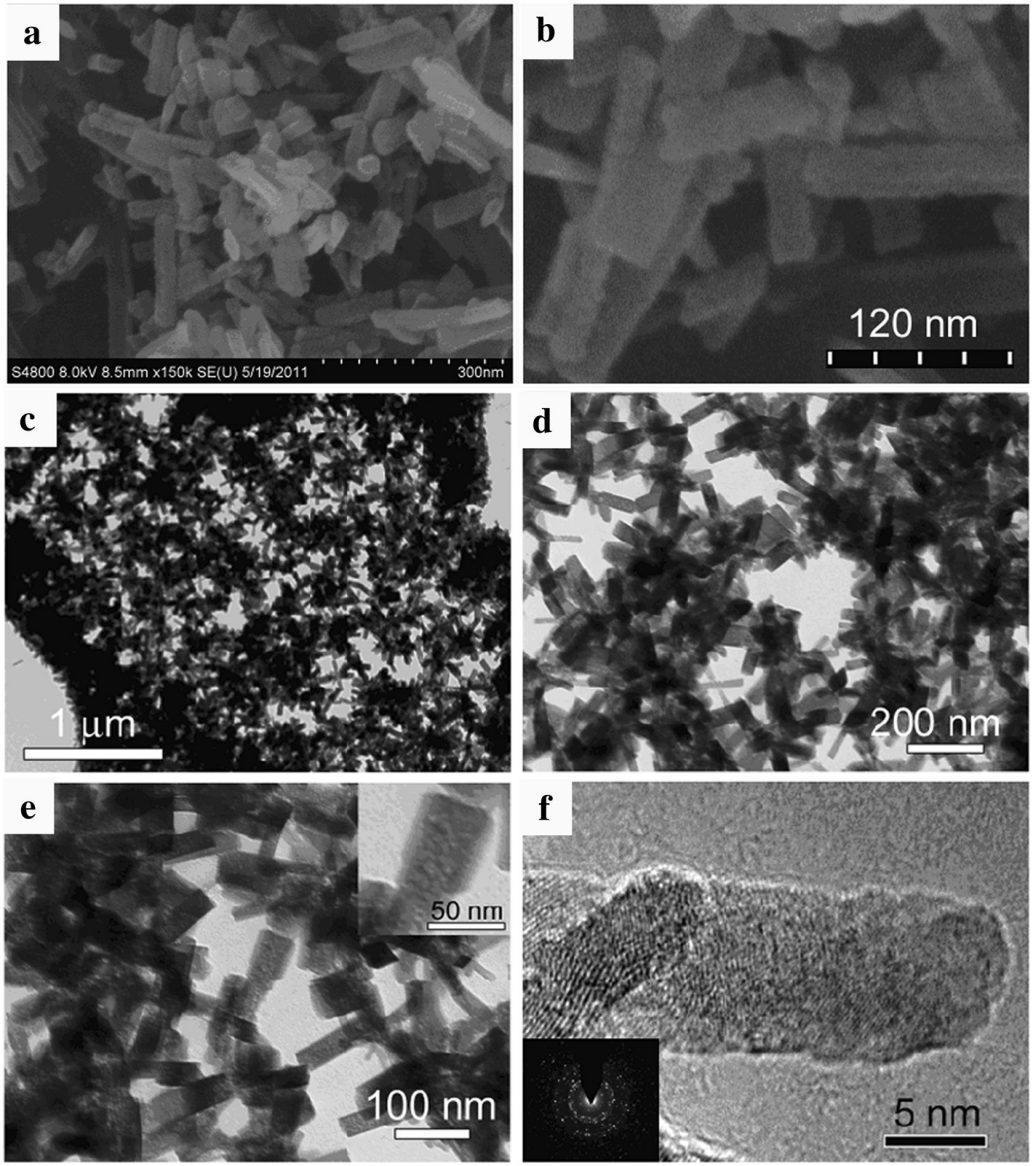
**Morphology of the cubic MnO nanorods obtained at 200°C for 24 h.** (**a**) Low-magnification and (**b**) high-magnification SEM images, (**c**, **d**, and **e**) TEM, and (**f**) HRTEM images. The inset in (**e**) is an enlarged TEM image, and the inset in (**f**) shows the SAED pattern of one single MnO nanorods.

The morphology of the as-prepared sample was examined by SEM and TEM. Figure 2a shows a typical SEM image of MnO nanorods synthesized at 200°C for 24 h, revealing that the product displays a uniform nanorod-like morphology. It can be observed that the nanorod is composed of small NPs, and the coarse surface of the nanorod can also be seen, as shown in Figure 2b. Figure 2c,d presents the TEM images of the unique MnO nanorods, showing that the lengths and diameters of the nanorods were *ca.* 100 to 200 nm and 20 to 30 nm, respectively. Figure 2e shows an enlarged TEM image, revealing the porous character of the nanorods. Figure 2f depicts an HRTEM image of one single nanorod, revealing that the obtained nanorod consists of small nanoparticle subunits. As shown in the inset of Figure 2f, the selected-area electron diffraction (SAED) pattern with polycrystalline-like diffraction also indicates that the nanorod is an ordered assembly of small nanocrystal subunits without crystallographic orientation, well consistent with the HRTEM results.

**Figure 3:**
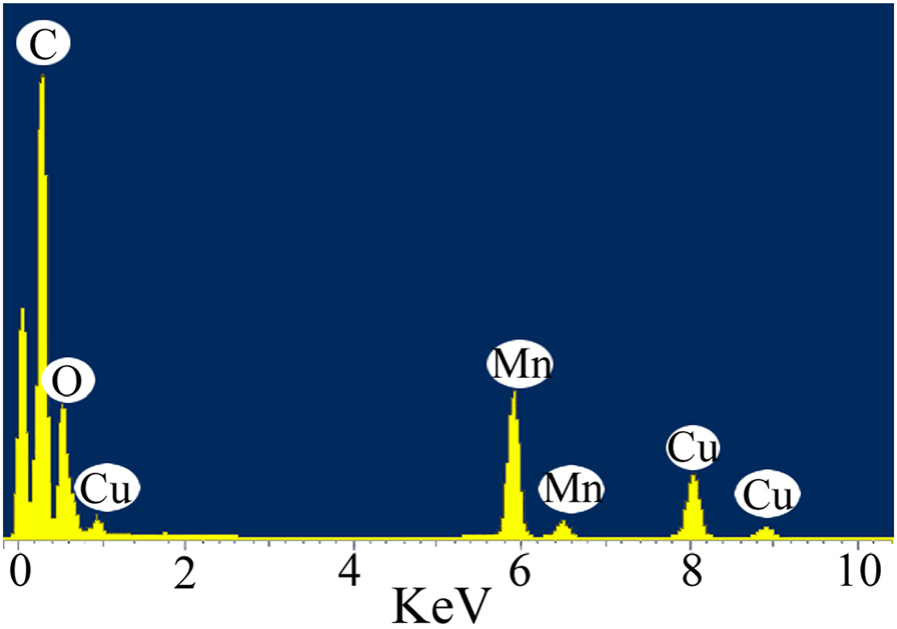
EDS spectroscopy of the as-prepared MnO nanorods.

The chemical composition of the as-prepared MnO nanorods was further confirmed by EDS analysis. The spectrum, taken from the center area of the nanorod, shows four strong signals of Mn, C, O, and Cu (Figure 3). The atomic ratio of Mn and O is about 1.02, indicating that the as-prepared nanorods are consist of high-purity MnO rather than other manganese oxides (e.g., Mn_2_O_3_, Mn_3_O_4_, and MnO_2_), in good agreement with the XRD results. The Cu and O may have resulted from the Cu gridding and C support membrane in the TEM observation.

**Figure 4:**
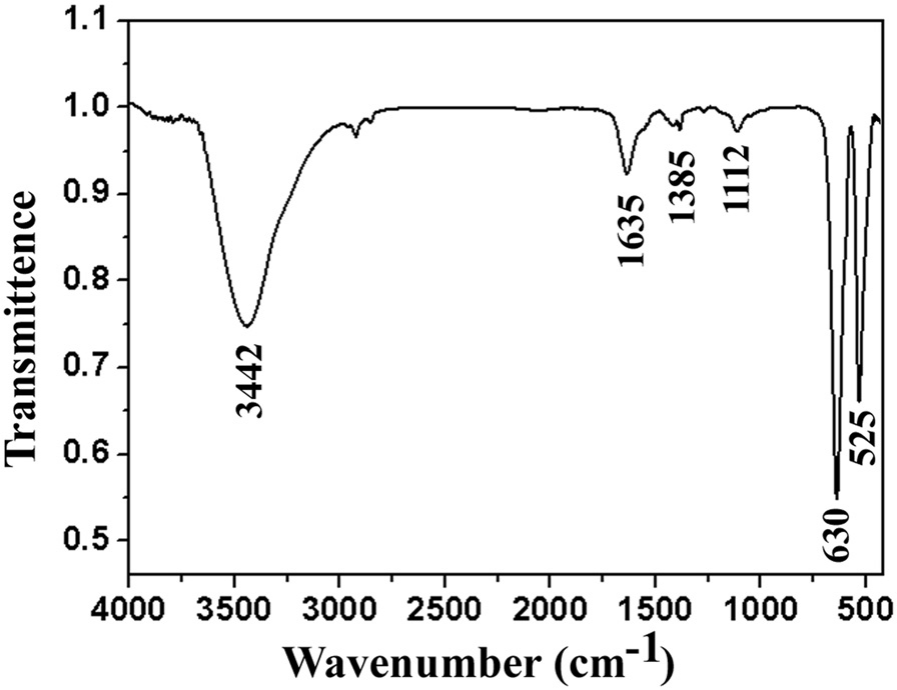
FTIR spectroscopy of the as-synthesized MnO nanorods.

The FTIR spectrum was further performed to substantiate the formation of MnO and the organic residue on the surface of MnO nanorods. As shown in Figure 4, two strong peaks at about 630 and 525 cm^−1^ arise from the stretching vibration of the Mn-O and Mn-O-Mn bonds [43], indicating the formation of MnO in the present work. In addition, strong absorptions at 3,442 cm^−1^ and weak absorptions around 2,800 to 3,000 cm^−1^ reveal the stretching vibrations of O-H and C-H, respectively. The absorption peak at 1,112 cm^−1^ corresponds to the C-OH stretching and OH bending vibrations, whereas the bands at 1,385, 1,580, and 1,636 cm^−1^ correspond to C-O (hydroxyl, ester, or ether) stretching and O-H bending vibrations [44,45]. These results indicate that some organic residues such as hydroxyl and carboxyl groups are present on the surface of the as-prepared MnO nanorods.

**Figure 5:**
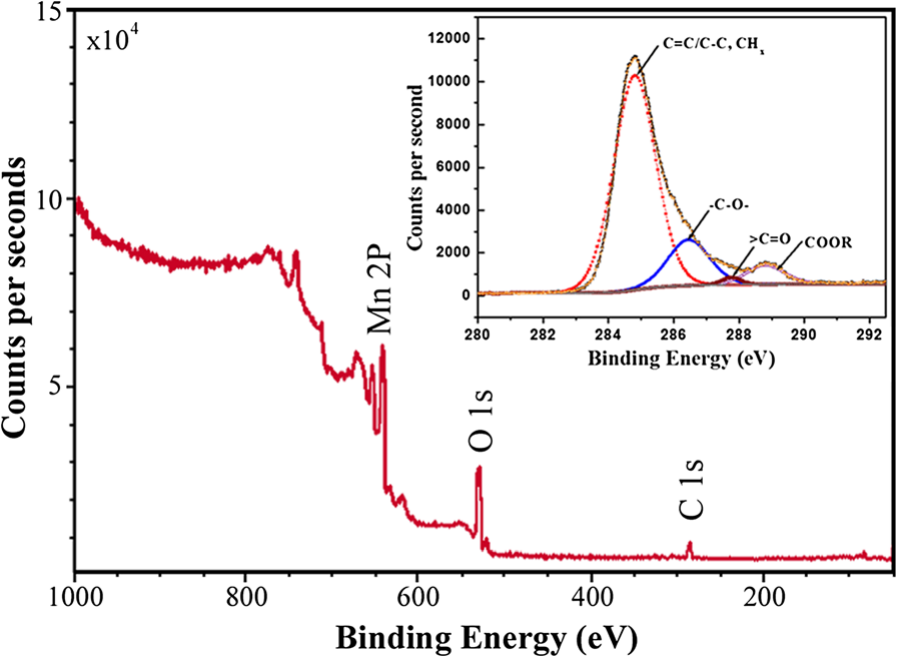
**XPS survey spectrum of the as-prepared MnO nanorods.** The inset shows the C 1*s* core-level spectrum and the peak fitting of the C 1*s* envelope.

The presence of the residue functionalities on the surface of the as-synthesize MnO nanorods was further characterized by XPS measurements. As shown in Figure 5, the survey spectrum shows the signals of Mn 2*p*, O 1*s*, and C 1*s*, indicating the presence of carbon element on the surface the nanorods. The presence of the organic groups was further confirmed by the C 1*s* spectrum. The inset in Figure 5 presents the C 1*s* core-level spectrum and the peak fitting of the C 1*s* envelope. Four signals at 284.8, 286.4, 287.6, and 288.8 eV were identified, which were attributed to carbon group (C = C/C-C, CH_*x*_), hydroxyl groups or ethers (−C-OR), carbonyl or quinone groups (>C = O), and carboxylic groups, esters, or lactones (−COOR), respectively. These results also reveal the presence of organic functional groups on the surface of the nanorods, in good agreement with the FTIR results.

**Figure 6:**
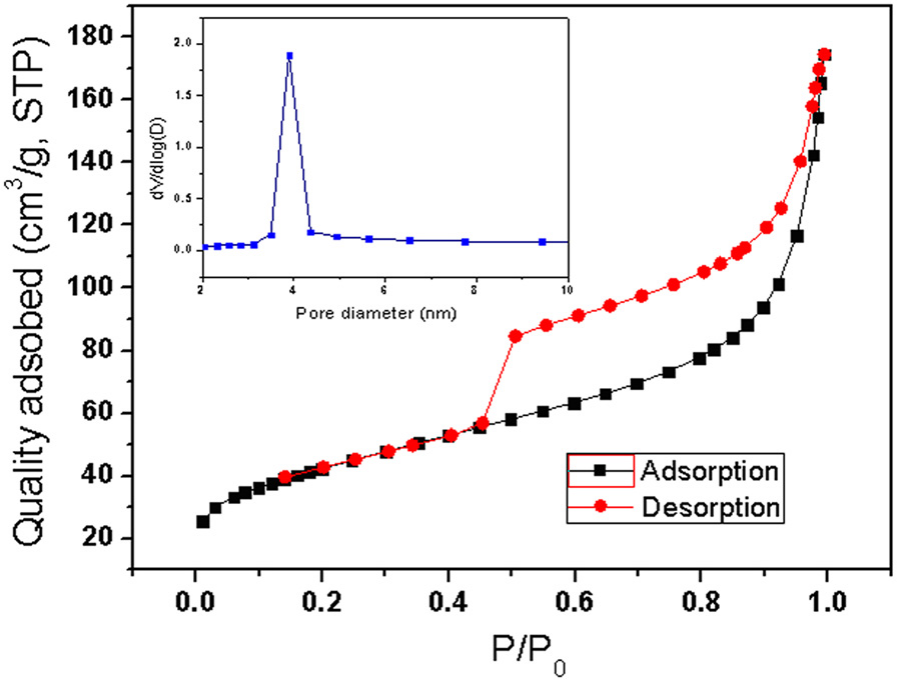
**N**_**2 **_**adsorption-desorption isotherms and pore size distribution curve of the MnO nanorods.**

The porous characteristic of the as-synthesized MnO nanorods was examined by nitrogen adsorption isotherm measurements. The specific surface area and pore size distribution (PSD) of the MnO nanorods were obtained from an analysis of the desorption branch of the isotherms using the density function theory. As shown in Figure 6, an isotherm is typical for a mesoporous material with a hysteresis loop at high partial pressures. According to the Brunauer-Emmett-Teller analysis, the as-synthesized MnO nanorods exhibited large specific surface area of *ca*. 153 m^2^ g^−1^ and pore volume of *ca*. 0.22 cm^3^ g^−1^. The inset in Figure 6 shows the Barrett-Joyner-Halenda PSD curve that was centered at *ca*. 3.9 nm, suggesting that the MnO nanorods possess uniform mesoporous structures.

**Figure 7:**
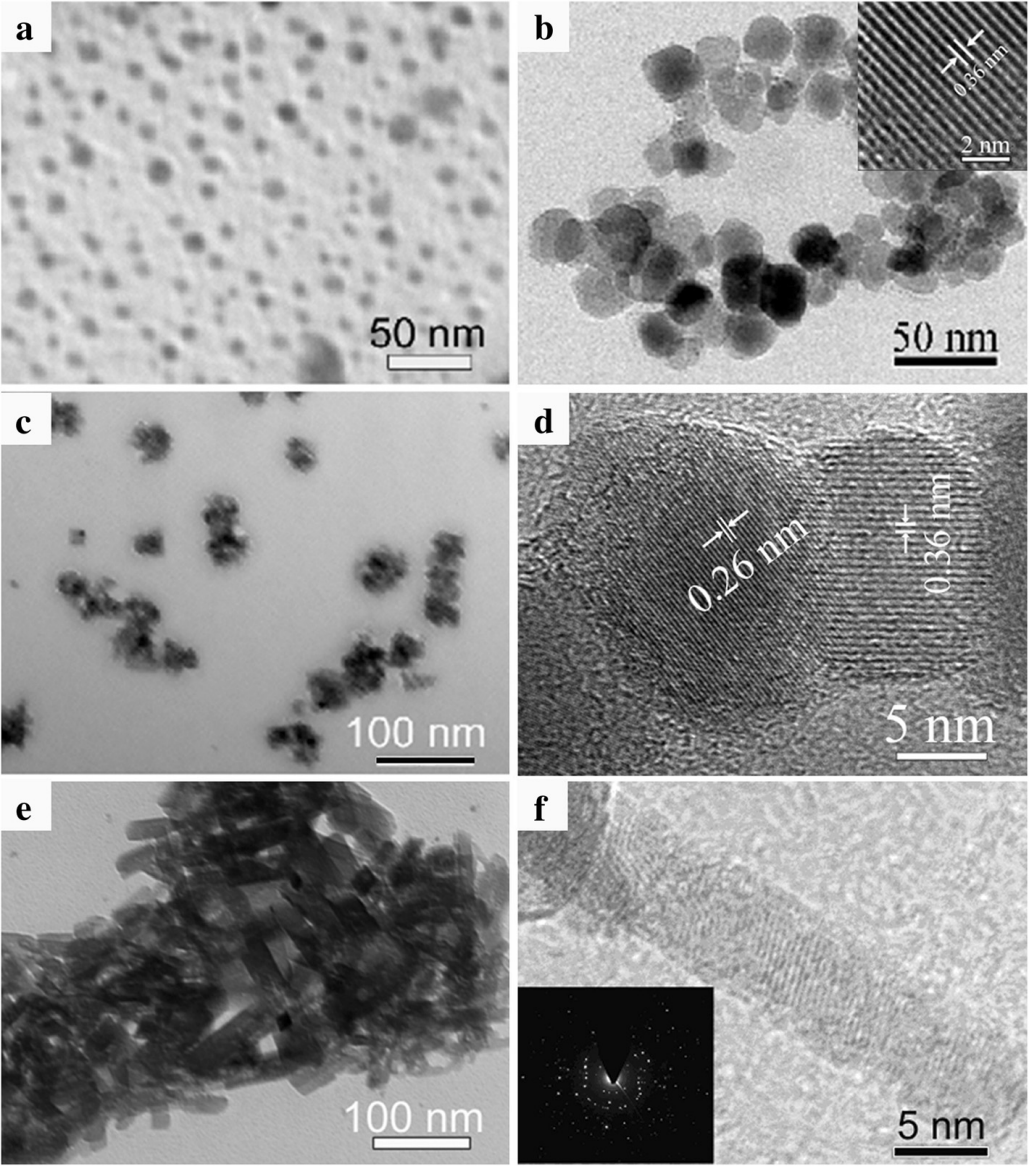
**TEM images of the as-prepared products at 200°C for different reaction times.** (**a**) 1 h, (**b**) 3 h, (**c** and **d**) 6 h, and (**e** and **f**) 12 h.

To investigate the formation mechanism of the MnO nanorods, a series of time-dependent experiments were carried out. As shown in Figure 7a, numerous amorphous manganese precursor NPs with size of *ca*. 5 to 6 nm were observed when the reaction was executed for 1 h. Figure 7b shows that larger NPs with size of *ca*. 20 to 30 nm were formed when the reaction time was increased to 3 h. The inset in Figure 7b reveals that the lattice fringe is *ca*. 0.36 nm, consistent with the *d*_012_ spacing for rhodochrosite MnCO_3_, indicating that the transformation from manganese precursor to MnCO_3_ happened in the earlier stage. When the reaction time was increased to 6 h, many nanorod-like particles could be obtained besides dispersed NPs (Figure 7c). It can also be seen that the nanorod-like products were formed by the self-assembly of small NPs. Figure 7d shows an HRTEM image taken from two adjacent NPs. The lattice fringes were found to be *ca*. 0.36 and 0.26 nm, corresponding to the *d*_102_ spacing for rhodochrosite MnCO_3_ and the *d*_111_ spacing for cubic MnO, respectively, suggesting that the transformation from MnCO_3_ to cubic MnO was incomplete within a short time. When the reaction time was further increased to 12 h, a large number of nanorods were formed (Figure 7e). Figure 7f shows an HRTEM image of one nanorod aggregated by small nanocrystals, and the boundary can be observed among the NPs. The SAED pattern in the inset of Figure 7f presents a polycrystalline character of the nanorods, indicating that the nanorod is of an ordered assembly of nanocrystals without crystallographic orientation.

**Figure 8:**
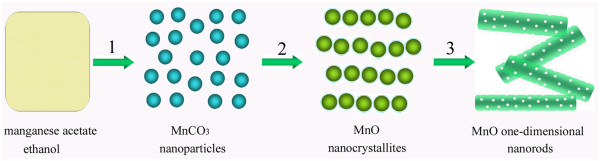
The possible formation mechanism of the MnO one-dimensional nanorods.

On the basis of the above experimental results, the possible formation mechanism of the MnO one-dimensional nanorods in the present work was proposed, as schematically illustrated in Figure 8. Firstly, the reaction between manganese acetate and ethanol results in the formation of certain alcohol acetate complexes, e.g., CH_3_COOMnOC_2_H_5_, accompanied with the nucleation and growth of amorphous precursor NPs, which are then transformed into MnCO_3_ nanocrystals (step 1). Secondly, with the increase of reaction time, the MnCO_3_ precursor is decomposed into MnO nanocrystallites (step 2). Meanwhile, the generated MnO nanocrystallites are capped by the short C-chain molecules forming oxide-organic hybrids, which act as build blocks to form novel MnO nanostructures. When two MnO building blocks come together, the capillary force between them facilitates the solvent removal and strengthens the agglomerate by van der Waals forces. Finally, with the increase of reaction time, directed self-assemblies of the oriented nanocrystallites and subsequent fusion lead to the formation of the MnO one-dimensional nanorods (step 3).

## Conclusions

In summary, uniform mesocrystalline MnO nanorods were prepared successfully by using manganese acetate and ethanol as starting materials. The as-synthesized MnO nanorods exhibited uniform morphology, large specific surface area, and narrow pore size distribution. The simple, cost-effective, and environmentally friendly synthesis can be scaled up to produce large quantities of porous MnO one-dimensional nanorods. Owing to their large specific surface area, the as-prepared MnO nanorods may have promising applications in energy storage, catalysis, and biomedical image. This method may also open a new avenue for the simple synthesis of porous functional materials with applications in the fields of energy and environment.

## Competing interests

The authors declare that they have no competing interests.

## Authors’ contributions

MZ synthesized the MnO nanorods and performed the structural characterizations. HZ carried out the BET experiments. XG and RX performed the XRD and FTIR experiments. YX, HD and XL discussed the possible formation mechanism of MnO nanorods. YL conceived of the study and revised the manuscript. All authors read and approved the final manuscript.
